# Involvement of ICU families in decisions: fine-tuning the partnership

**DOI:** 10.1186/s13613-014-0037-5

**Published:** 2014-11-30

**Authors:** Elie Azoulay, Marine Chaize, Nancy Kentish-Barnes

**Affiliations:** 1 Medical Intensive Care Unit, Hôpital Saint-Louis, ECSTRA team, Biostatistics and clinical epidemiology, UMR 1153 (Center of Epidemiology and Biostatistic Sorbonne Paris Cité, CRESS), INSERM, Paris Diderot Sorbonne University, Paris, France

**Keywords:** Information, Communication, End of life, Bereavement, Randomized controlled trials

## Abstract

Families of patients are not simple visitors to the ICU. They have just been separated from a loved one, often someone they live with, either abruptly or, in nearly half the cases, because a chronic condition has suddenly worsened. They must cope with a serious illness of a loved one, while having to adapt to the unfamiliar and intimidating ICU environment. In many cases, the outcome of the critical illness is uncertain, a situation that causes considerable distress to the relatives. As shown by our research group and others, families exhibit symptoms of anxiety (70%) and depression (35%) in the first few days after admission, as well as symptoms of stress (33%) and difficulty understanding the information delivered by the healthcare staff (50%). Furthermore, relatives of patients who die in the ICU are at risk for psychiatric syndromes such as generalized anxiety, panic attacks, depression, and posttraumatic stress syndrome. In this setting of psychological distress, families are asked to consider sharing in healthcare decisions about their loved one in the ICU. This article aims to foster the debate about the shared decision-making process. We have three objectives: to transcend the overly simplistic position that opposes paternalism and autonomy, to build a view founded only on an evaluation of actual practice and experience in the field, and to keep the focus squarely on the patient. Families want information and communication time from the staff. Nurses and physicians need to understand that families can share in decisions only if the entire ICU staff actively promotes family involvement and, of course, if the family wants to participate in all or part of the decision-making process.

## Review

### Introduction

Advances in medicine have considerably improved life expectancy, in part by allowing patients to survive critical illnesses. In some countries, patients who want to participate actively in decisions about their healthcare are strongly encouraged to do so. In the ICU, however, and in many areas of acute care, patients often exhibit cognitive impairments that prevent them from providing informed consent or participating in decisions designed to avoid nonbeneficial interventions or to determine which treatment option provides the best risk-benefit trade-off [1],[2]. Consequently, their families are often asked to speak for them when difficult decisions must be made. These decisions hinge around complex and potentially distressing issues such as the duty to preserve life, the right to a peaceful and dignified death, and the balance between length of life and quality of life [3].

When the patient cannot communicate, the private haven of the doctor-patient relationship is replaced by intercommunication between the entire medical staff and several of the patient's relatives. Involving the family in the decision-making process raises complex challenges. Not all patients discuss their wishes with their family. The family members may have difficulty shifting their perspective from what *they* want to what they believe the *patient* wants. Finally, some family members may consciously or subconsciously make decisions without a clear understanding of what is at stake [4] or defend their own best interests rather than the patient's [5]-[8], without making reference to possible interference from the information provided on the Internet [9].

Unfortunately, the debate about sharing decisions with families of the ICU patient was long confined to a confrontation between paternalism and autonomy [10]. These two models of the doctor-patient relationship are obsolete in our opinion, as are the reasons put forward to explain the differences in medical decisions that occur across countries [11]-[13].

Our objective with this review article is to strengthen the case for involving families in the decision-making process in the ICU. We will draw only from evidence supplied by epidemiological studies and practice evaluation surveys done in ICUs in France and elsewhere. Similarly, the concepts that we suggest are founded on clinical experience and on an analysis of complex situations.

### The doctor-patient relationship: theoretical models

The doctor-patient relationship shifted from the paternalistic to the autonomy model in the 1970s [14]. According to the paternalistic model, the patient is passive and unable to make wise choices. The physician knows best and protects the patient (acting as a guardian) from making mistakes [15]. The patient trusts the doctor, who exercises control, makes all the decisions, and establishes priorities regarding the management plan, usually based on the scientific evidence. The paternalistic model was called into question when studies showed considerable variability in practice patterns within small geographic areas whose populations were similar in terms of health status and resources, as well as for health conditions whose management is laid out in clinical guidelines [16]. Subsequent studies established that this variability was due to the influence on decision-making of individual factors of such training [17] and cultural and religious values [13]. In addition, when several treatments are available, the best trade-off between benefits and risks depends in large part on the patient's perceptions and consequently cannot be determined by the physician alone. Similarly, whether treatment limitation decisions are appropriate depends in part on whether the quality of life that would be obtained should the patient survive with full-code treatment is acceptable to the patient. Studies establishing the role for subjective factors made a strong case for patients (or their families) playing an active role in choosing which types of healthcare they received. Thus, the autonomy model was developed as the antithesis of the paternalistic model.

The autonomy model gained popularity in the 1980s in North America and other English-speaking countries. In this model, the doctor is a consultant who informs the patients and families of the diagnosis and treatment options, describes the risks and advantages of each option, and listens to the patients to identify their preferences. Ultimately, the decision about which option to use lies with the patient or family. In this model, patients and families are assumed to have an objective understanding of scientific data that allows them to make informed choices without guidance. However, this model was rapidly recognized to have a number of shortcomings. Patients and families do not always gain a clear understanding of the scientific problems involved in decisions about healthcare [5],[18]-[20]. They may be coping with severe pain, other symptoms of serious illness, or marked emotional distress, which may limit their ability to step back and gain an objective perspective on their health situation [20]. Family members acting as surrogates may experience difficulty in separating what they feel is best from what they believe the patient would think is best [21]-[24]. When a critical illness occurs, complex decisions must be made under considerable time pressure, placing an additional constraint on the patient and family. These obstacles to full patient autonomy prompted the development of a model in which decision-making is shared by the patient or family and by the physician.

In the shared decision model, patients or families and physicians work together as partners to make healthcare decisions [12],[25]. Importantly, establishing a sound partnership requires careful attention to effective communication. The 2003 international consensus statement on managing ICU patients at the end of life strongly recommends the shared decision model as a dynamic strategy that allows adaptation to each specific situation [26]. The physicians' goal in the shared decision model is to empower families by helping them to understand what is at stake and, if they so wish, to share in the decision-making process. Conversations are held to allow the healthcare staff to supply scientific information and the families to explain the values and preferences of the patient, vent their emotions, and voice their concerns. During these conversations, the healthcare workers can provide emotional support to the patient and families, verify that the information supplied has been understood, indicate which course of action they deem best and why, and help families identify psychological and emotional barriers to objective decision-making. Furthermore, healthcare workers trained in using the shared decision model have learned to identify not only emotional and cognitive factors affecting the decision-making of patients and families but also their personal preferences and values, which may color their judgment in a way that differs from the patient's preferences and values [27]-[29].

In theory, the shared decision model combines the advantages of the paternalistic and autonomy models, by allowing patients or families to contribute to the extent that they can, while receiving guidance from the healthcare team. However, in everyday practice, sharing decisions has proved difficult to accomplish, particularly for ICU patients who are represented by their families. In a study from Seattle in the US, ten criteria defining shared decision-making were developed [30]. These criteria were then used to evaluate audiotapes of end-of-life family conferences in four ICUs. The results showed that only 1 of 51 decisions met all ten criteria, and the mean value on a 10-point shared decision-making scale (Table 1) was only 6.1 ± 1.8 [30]. These results establish the existence of obstacles to shared decision-making.

**Table 1:** **Relationship between coded physician behaviors and shared decision-making (adapted from White**[30]**and Charles**[25]**)**

**Dimension of shared decision-making**	**Coded physician behaviors**	**Practical example**
Providing medical information	1. Discuss the nature of the decision.	1. Last week, we told you that he had severe cardiac insufficiency. But now, the stroke makes his/her odds of immediate survival very dismal
	What is the essential clinical issue we are addressing?	
	2. Describe treatment alternatives	2. Instead of performing the tracheostomy now, we could extubate him/her and see if it works
	What are the clinical reasonable choices?	
	3. Discuss the pro and cons of the choices	3. Each strategy has strengths and weaknesses, and it is important to balance each strategies
	4. Discuss uncertainty	4. We are beginning new antibiotics for this VAP, but his/her status may worsen
	What is the likelihood of success of treatment?	
	5. Assess family understanding	5. Check whether family has actually grasped which decision is being made and what are its consequences
	Is the family an informed participant with a working understanding of the decision	
Eliciting patient's values and preferences	6. Elicit patient's values and preferences	6. As you know him/her well since a long time, could you please tell us what he would have said if he/she were sharing this discussion with us
	What is known about patient's preferences and values?	
Exploring the family's preferred role in decision-making	7. Discuss the family's role in decision-making	7. Family should be offered a role in decision-making even if some will decline
	8. Assess the need for input from others	8. Is the primary physician, the general practitioner, or any close friend that the family would like to consult?
	Is there anyone else the family would like to consult?	
Deliberating and decision-making	9. Explore the context of the decision	9. Terminal weaning with likely immediate death versus decision of no escalation of treatment
	How will the decision affect the patient's life?	
	10. Elicit the family's opinion about the treatment decision	10. The decision is that we are not going back to surgery as it is likely to fail and to prolong unnecessary suffering. What do you think of this decision?
	What does the family think is the most appropriate decision for the patient	

Obstacles to shared decision-making include difficulties experienced by patients and families in understanding the information they receive [18],[19] and emotional distress of sufficient magnitude to impair the patient's or family's ability to make decisions that are in the patient's best interests [18]-[20]. In addition, because of organizational factors or of the rapid progression of a life-threatening illness, the healthcare team may not have enough time to hold the conversations required to build trust and to get to know the patient and family. The patient or family also needs time to form an opinion about the healthcare team. Furthermore, information about treatment options is usually available in terms of means for patient populations, whereas individual patients are interested only in the effects of the treatment on themselves. Shared decision-making requires that the healthcare workers tailor the information and discussion to the background, wishes, and experience of each patient or family. Shared decision-making assumes that the ICU staff can convey their knowledge in simple and readily understandable words that nevertheless allow the patient or family to grasp the nuances and consequences of each decision. There is a convincing evidence that the manner in which information is conveyed to patients strongly influences the nature of the decisions made [31]. For instance, in a study of patients with lung cancer, patients were asked to choose between surgery and radiation therapy, which were considered equivalent in terms of effectiveness and safety. When communicating the results of these two strategies to the patients, the statistics were framed in two different ways, with emphasis either on survival or on mortality. With the survival framing, 18% of patients chose radiation therapy, compared to 44% with the mortality framing [31]. Thus, there is no doubt that physicians consciously or subconsciously influence the choices made by their patients. This example illustrates the tight links between patient autonomy and the quality and objectivity of the information supplied to the patient. As emphasized further in this article, in addition to the information itself, a crucial point is communication with families, including a willingness to listen and to engage in meaningful conversations.

In most of the satisfaction scales developed for the ICU, participation in care, discussions, and decisions is listed among the factors that increase family satisfaction [32]-[34]. In Canada, where surrogates make decisions for the patients they represent, surrogate satisfaction with the decision-making process was found to increase with the quality of the information received [32]. On both sides of the Atlantic, participation of families in decisions is associated with greater satisfaction [32],[33],[35]-[37]. When 8,000 residents of France were surveyed, 90% of responses indicated a desire to have family members share in decisions about their care should they require ICU admission [38]. In France and in other countries, the overwhelming majority of healthcare providers are willing to have families share in medical decisions. However, an unwillingness of family members to share in decisions must be honored. The delegation of decisional authority is after all an autonomous decision. In a French study, more than half of the families did not want to share in medical decisions [7], and the number of relatives willing to share decisions was even lower among families that were satisfied with the quality of the information they received. Sharing in decisions can result in a substantial burden being placed on the families. Thus, in families of patients involved in end-of-life medical decisions, the risk of posttraumatic stress symptoms was increased 3 months after the death [39]. However, the staff should seek to determine whether the unwillingness to share in decisions is related to psychological distress, which may be amenable to alleviation, or to poor understanding of the situation, requiring additional information and communication efforts.

Specific challenges arise when patients die in the ICU. Families of patients who die in the ICU have higher rates of anxiety and depression symptoms [40],[41], sleepiness [20], psychiatric syndromes (e.g., panic attacks, generalized anxiety, and pathological grief reaction) [42], and posttraumatic stress [39]. They report overwhelming guilt and regrets about the circumstances of their loved one's death in the ICU [6],[43]-[46]. Clearly, the extreme vulnerability of these families warrants intensified communication efforts [47]. In a large cohort study, the death of a spouse led to severe distress in the surviving spouse, who had a nearly 20% increase in the risk of death [48]. These studies emphasize that families entering the grieving process need to be accompanied by communication strategies that combine empathy, compassion, listening, attending, and palliation. Similarly, ICU nurses and physicians can experience considerable distress when patients die in the ICU. Thus, the management of dying patients and their families are major determinants of conflicts in the ICU [49], as well as of burnout syndrome [50],[51]. Clearly, all those involved in end-of-life situations need help and support. Preventing burnout syndrome and conflicts with families or within the staff are strong reasons to work actively on communication strategies that support families, with the primary goal of easing the grieving process and of diminishing the risk of pathological grieving. Clinicians should be aware of their duty when managing ICU conflicts and developing strategies aimed at reducing burnout.

Thus, although shared decision-making is clearly preferred today over other models, we are still learning about how to best respect the patient's autonomy (often via the family) while providing the appropriate level of guidance. Whereas the paternalism and autonomy models had fairly simple rules designed to confine the patient-physician interaction within narrow boundaries, shared decision-making is emerging as a spectrum of interaction patterns whose characteristics are specific to each patient or family. Shared decision-making must be fine-tuned to each case. If healthcare workers are to use the shared decision model, they must be highly skilled in communicating with the patient or family, as well as clearly aware of their personal sources of bias.

### Fine-tuning shared decision-making

ICU teams must develop a staff-family relationship that is not only proactive but also context-sensitive [52]-[54]. It should also be reminded that the decision-making process is not a ‘single shot decision’. Instead, most decisions require time to adapt, cope, and sometimes negotiate. For the sake of simplicity, we present the process of making a decision as a simplicity and binary event; however, we do recognize that this is not the reality.

The shared decision model has clear limitations but can be improved by directing attention to the needs of each specific situation [55]. In particular, the nature of healthcare decisions for ICU patients varies widely, and an important step is the identification of decisions for which offering a role to the family is not only an ethical obligation but also likely to benefit all those involved. These decisions are often characterized by either uncertainty regarding the outcome of an intervention [56] (e.g., the patient may survive with or without severe cognitive deficiencies) or the superiority of one intervention over another [57] (two interventions with similar effectiveness and safety profiles are available). In both situations, moral or religious beliefs and personal values play a major role in determining which is the best choice. There is a crucial need for shared decision-making, even when the outcome is easy to predict. When recovery of preexisting physical and cognitive function is almost certain to occur, or when a low-risk intervention is mandatory (e.g., insertion of a parenteral nutrition catheter), *information* during a conversation, with or without the help of a printed information sheet, helps to improve comprehension and to decrease anxiety and depression symptoms in the family members [56]. This strategy also empowers the relatives for an accurate decision-making process. When a high-risk intervention is mandatory on an emergency basis (e.g., blood transfusion in a patient with hemorrhagic shock due to stab wounds), the need for immediate treatment overrides the duty to provide information first. When death is almost certain to be the outcome regardless of whether life-supporting interventions are used, the focus of communication with the family should be on *palliative strategies* designed to maximize patient comfort and on negotiation with the family members to minimize any feelings of guilt [58],[59]. Midway between these two situations, the outcome is difficult to predict and specific communication efforts are needed to help the family cope with this uncertainty (e.g., *n*th surgical procedure or additional chemotherapy course) [60]. The ICU staff must use a considerate and sensitive approach to help the family understand that death or disability is a distinct possibility but that there is hope for recovery with a good quality of life. Finally, when two interventions similar in terms of effectiveness and safety are available, the family's input about the patient's preferences and values is crucial.

The patient autonomy model was initially described as the physician providing impartial information then leaving decisional authority entirely to the patient or family. Because the patient and family may experience difficulties understanding the scientific issues involved or preventing their emotions from affecting their ability to make decisions that are in their best interest, a version of the autonomy model known as neopaternalism was developed [61]. Neopaternalism adjusts the concept of autonomy to the reality of patient and family capabilities by having the healthcare staff give a clear description of their personal opinions, based on their experience and on what they believe is in the patient's best interests [61]. In the shared decision model, such as description, can serve as a useful starting point for discussions with patients or families.

Families may be given the opportunity to exercise their right to autonomy (formal autonomy) yet be prevented by bounded cognition from making the decisions they deem in the best interests of the patient (effective autonomy). Bounded cognition is the set of cognitive, information, and time limitations that affect the rationality of decision-making by both families and healthcare workers. A systematic analysis of these limitations (debiasing [31]) may help to circumvent them. Specific efforts must be made to identify cognitive and emotional influences that may bias the decisions of both the family and the staff. For instance, a bias toward overconfidence in decisions can be counterbalanced by a description of alternative outcomes. Staff members must be aware of experiences and emotions that may affect the way they frame the information they give to families. Families can be helped to understand how their fears, past experiences, or current state of emotional distress may influence their decisions in a way that runs counter to the patient's values and wishes.

Regardless of the clinical, social, and psychological situation, families may decide to delegate their decisional authority to the ICU staff. This decision should be respected. After all, a decision to delegate is also an expression of autonomy. Families who delegate their decisional authority must be kept informed of changes in the patient's situation and offered the possibility of entering the decision-making process at any time if they so wish.

Thus, instead of a power-based description of the physician-patient (family) relationship as either paternalistic or autonomous, this relationship is best viewed as a complex partnership in which power has no place. In the shared decision-making model, the healthcare workers bring their scientific knowledge and experience to the partnership, whereas the family members bring their familiarity with the patient's values and wishes, symptoms, and quality of life. Both the staff and the family strive to gain awareness of factors that may bias their decisions. The staff presents the options with their expected outcomes and both the staff and family indicate which option they prefer and why. Careful attention is given to ensure that the family understands what is at stake and has a clear grasp of any uncertainty regarding the outcome. The staff must be aware that delivering fully objective information may be nearly impossible.

Clearly, the development of the family-staff partnership requires specific and intensive efforts. A prerequisite is good teamwork within the ICU. Actions likely to foster a healthy family-staff partnership are outlined below.

## Conclusions

### Ten key points to improve family care in the ICU (Table 2)

**Table 2:** Ten key points to improve family care in the ICU

**Ten key points**
The nurse-physician liaison pair
Regular debriefing meetings attended by both physicians and nurses
Sharing decisions between physicians and nurses (decision-making meetings)
Moving from information to communication
Opening the ICU visiting hours
Informal and brief conversation with the family at ICU admission
Formal meeting on the third ICU day
The end-of-life conference
The ICU discharge visit
Evaluating information and communication practices and teaching communication skills to healthcare workers

1) The nurse-physician liaison pair

We strongly recommend that the nurse and physician inform the family together, if at all possible. In addition to having didactic advantages, this approach ensures that the ICU team is perceived as united and that families do not receive contradictory information. It also highlights the valuable role for the nurse in the communication process and provides a written record of each conversation [62].

Along the same line, patient comfort should be evaluated by the liaison pair, to the extent possible, most notably at the end of life in the ICU. Having the physicians and nurses work together to evaluate pain at the end of life decreases the rate of associated conflicts [49],[63],[64].

2) Regular debriefing meetings attended by both physicians and nurses

Debriefing meetings provide physicians and nurses with a unique opportunity to exchange their points of view about a specific patient, a family member, or a complex medical case. Importantly, these meetings allow the participants to openly share their perhaps very personal perceptions of a person or situation and also to gain awareness of their own sources of bias. They are associated with greater satisfaction among families and nurses [35],[51] and with lower within-team conflict rates [49]. Ideally, each team should meet once a week, and physicians on night duty should hold meaningful discussions with the night shift nurses.

3) Sharing decisions between physicians and nurses (decision-making meetings)

These meetings are also known as ethics meetings. They are considered as a prelude to possible treatment limitation decisions. The entire history of the patient is reviewed, and the irreversible nature of the current medical situation is confirmed. These meetings are the mark of a collegial decision-making process, which is far more common in France than in other countries [65]. When making decisions, physicians tend to rely on statistical data (to identify futility) and nurses on qualitative data (pain, anxiety, and quality of life). These differences underline the complementarity of input from nurses and physicians in the decision-making process [66].

4) Moving from information to communication

Among the key features of communication, listening alleviates distress in families [29],[58],[59],[61],[67]. In end-of-life conferences, families who spend more time describing their concerns and verbalizing their emotions and difficulties have higher levels of satisfaction and a lower risk of pathological grieving [58],[67],[68]. In addition, letting families choose the time of the conference so that they can arrange for a larger number of members to attend (i.e., parents or children) substantially improves the quality of communication.

5) Opening the ICU visiting hours

We will touch briefly on this topic, which would deserve an extensive review. We suggest that ICU teams keep an open mind and evaluate this issue in a nondogmatic manner. Five key points can be borne in mind when considering visiting hour policies [69]. (a) Suggesting and discussing new options works better than imposing a change. A good way to open the debate is to have a task force of physicians and nurses discuss the pros and cons of each option. (b) Evaluate openness to a change in policy among healthcare workers, families, and patients. (c) A 24-h visiting policy is not always the best option. For instance, in an ICU that allows visits during two 2-h periods, allowing visits from noon to 11 pm would constitute a major improvement. Further broadening of the visiting policy is likely to occur later on. (d) The visiting time is not the same as the information time. A more liberal visiting policy requires increased efforts to organize the delivery of information (i.e., start a round of the families at 2:30 pm). (e) Finally, the visiting policy and organization of information delivery should be explained clearly in the family information leaflet [18],[70],[71].

6) Informal and brief conversation with the family at ICU admission [5]

When a new patient arrives, the ICU staff is often busy settling the patient in, reading the medical chart, and performing life-supporting interventions. Nevertheless, a resident or senior physician should take a few minutes to *actively approach* the family and say a few words. Useful information at this stage is that the patient is being taken care of, that the symptoms are being analyzed although it is still too early for a diagnosis, that life-supporting measures (e.g., antibiotics, dialysis, ventilation) are being taken, and that more will be known about the situation on the next day. A team that knows how to gain the family's confidence at the time of admission is more likely to succeed in building a trust-based relationship that will persist throughout the ICU stay [72]. The impact of the information leaflet can also be enhanced by this brief information [70] that could definitely discourage the use of the Internet [9] and to patient-targeted googling [73].

7) Formal meeting on the third ICU day [74],[75]

This meeting is crucial to the organization of information and to the development of the communication strategy. In our experience, the day 3 meeting is a powerful tool. On day 3, the ICU workers actively approach the family to organize a formal meeting for the next day or when the family can arrange for several members to attend. The format is formal, and the meeting must occur in a quiet place. Pagers and phones should be turned off. Staff members should identify themselves. The family members should then be asked to say, in their own words, what they know about the situation, and any errors in communication should be corrected. The family should be able to understand the prognosis and to detect the signs indicating that the treatment is working (e.g., awakening, resumption of urine output, discontinuation of vasopressors, and/or extubation). Importantly, the physicians and nurses must tell the family that they are available (together) to answer any subsequent questions and should arrange for another meeting 48 h later or earlier if needed.

8) The end-of-life conference [27],[29],[58],[67],[68]

The end-of-life conference applies the day 3 meeting concept to patients who die in the ICU. It was first described by Randall Curtis and the Seattle group then validated by the Famiréa group in France [27],[29],[58],[67],[68]. Major objectives for the team are to acknowledge the value of the family's input, to check that the family members understand the information they have received given so far, to spend much more time *listening* than speaking, to indicate clearly that the best interests of the patient are at the heart of everything that is being said (the focus is on the patient), and to invite the family to ask questions [63]. At completion of the end-of-life conference, we give the family a brochure derived from the SPARADRAP brochure developed by a multidisciplinary pediatric task force (www.sparadrap.fr).

9) The ICU discharge visit

Both patients and families are vulnerable at discharge from the ICU. The high intensity of care and numerous staff-family interactions in the ICU must be left behind, which may generate feelings of abandonment. Anxiety may result from the need to deal with an unknown department, new nurses, and new policies for patient management and information. A study by the Famiréa group is currently evaluating the ICU discharge visit. This visit is a valuable opportunity for reassuring the patient and family and providing information, for instance about the common occurrence of symptoms such as nightmares and the expectation that these symptoms will resolve within a few weeks. Handing the ICU discharge summary to the patient and suggesting a visit to the usual physician should the post-ICU symptoms persist are additional ways to provide support.

10) Evaluating information and communication practices and teaching communication skills to healthcare workers

This step must become a standard practice. Evaluating our communication practices allows us to make further progress. Teaching communication skills to nurses and physicians is the key to improve our practices [76]. Changes for patients and families in the short and medium terms must be measured.

Finally, if you remain unconvinced that families are more than simple visitors to the ICU, ask yourself these three questions [77]:

1. When a patient is unable to communicate, who is most likely to have the knowledge needed to ensure that the management strategy respects the patient's values, preferences, and choices: you or the family?

In all likelihood, the family is in the best position to defend the patient's *best interests*. Obviously, these considerations do not apply to patients who do not want their families to receive information about their health situation. Furthermore, if the various members of the healthcare team feel the family's description of the choices the patient would have made is biased by a conflict of interest, then the decisions must focus on the patient's best interests, and advice from an independent consultant must be obtained and recorded in the medical chart.

2. Who is most deeply affected on a personal level by the results of the medical decisions?

Family members experience severe distress when a loved one is suddenly admitted to the ICU. They often use terms such as ‘cruel’ or ‘violent’ to describe their suffering and feelings of emptiness. Although they are often unable to make decisions alone, and although clearly they must not be made to bear the burden of responsibility and guilt associated with major decisions, they must be involved in the process that will allow them to understand why a given decision is in the best interests of their loved one. In both curative care and palliative care, only daily information and effective communication can allow families to understand and accept that the decision made collegially by the ICU team is the best possible one. The decision remains based on medical considerations, except in the rare cases where the patient has repeatedly stated an unwillingness to become dependent on a life-supporting technique or to witness his or her own decline [78]. In this case, the decision belongs to the patient and is either taken by the patient or based on advance directives (very rarely) or statements made to the family, usual doctor, or referring physician.

3. If the patient dies, who will experience the most distress?

The burden is of course greatest for the family. Family members are at risk for pathological grieving, [45],[79]-[82], depression [42], posttraumatic stress disorder, [39],[83], and death [48].

## Abbreviation

ICU: intensive care unit

## Competing interests

The authors declare that they have no competing interests.

## Authors’ contributions

EA has drafted the review article. MC and NKB have reviewed and made significant suggestions and changes in the manuscript. All authors read and approved the final manuscript.
